# Re: “impact of aminoglycosides on survival rate and renal outcomes in patients with urosepsis: a multicenter retrospective study”

**DOI:** 10.1186/s13613-025-01504-5

**Published:** 2025-11-18

**Authors:** Min Li, Min Xu

**Affiliations:** The Affiliated Jiangning Hospital of Nanjing Medical University, Nanjing, 211166 China

Dear Editor,

We read with great interest the recent study examining the renal and survival outcomes associated with aminoglycoside (AG) use in critically ill patients with urosepsis [1]. While the authors provide valuable insight into this important question, we are concerned that the study’s conclusion regarding renal safety may be premature, given the limitations of its nephrotoxicity assessment strategy.

## Is serum creatinine enough??

The authors rely primarily on changes in serum creatinine (SCr) and the MAKE30 composite endpoint to define renal injury. Yet SCr is a notoriously delayed and insensitive marker of acute kidney injury (AKI), particularly in critically ill patients. It is well established that SCr can remain normal until 24–48 h after significant renal tubular damage, and is highly influenced by non-renal factors such as volume status, age, and muscle mass [2]. Could this have led to systematic under-detection of early or mild AG-induced nephrotoxicity?

## Why was urine output ignored??

The KDIGO criteria for AKI diagnosis explicitly require both serum creatinine and urine output. Oliguria often precedes SCr elevation and is a critical early warning sign of renal dysfunction. Yet the authors provide no data on urine output or oliguria duration. Without this, how can we be confident that the study adequately captured the full AKI spectrum?

Furthermore, KDIGO staging (1–3), which reflects severity of injury, is not reported. Identifying even mild AKI (Stage 1), particularly in patients with AG exposure, is clinically important, given its known association with longer hospital stays and increased mortality [3]. Does the absence of KDIGO staging conceal important dose-response or severity patterns?

## Where Are the Biomarkers?

Modern nephrology has moved beyond serum creatinine alone. A growing body of literature supports the use of tubular injury biomarkers such as NGAL (neutrophil gelatinase-associated lipocalin), KIM-1 (kidney injury molecule-1), and IL-18 to detect subclinical nephrotoxicity—particularly in the setting of aminoglycoside exposure [4]. Shouldn’t a study evaluating AG-associated kidney injury consider these more sensitive and pathophysiologically relevant tools?

One study in ICU patients found that NGAL levels rose significantly within hours of AG administration, even in the absence of SCr changes [5]. Could the omission of such markers in the present study have masked clinically relevant injury?

## The risk of overinterpreting renal safety

By concluding that AGs are not associated with increased nephrotoxicity, the study may inadvertently promote overconfident clinical use of AGs, especially in settings lacking routine therapeutic drug monitoring (TDM) or early injury surveillance. Given AGs’ well-established nephrotoxic potential, such reassurance should be grounded in comprehensive and sensitive renal assessment, not just conventional endpoints.

## Conclusion

While we appreciate the effort to clarify the safety of AGs in urosepsis, the use of crude renal endpoints—without urine output, KDIGO staging, or biomarker integration—renders the study’s renal safety conclusion incomplete at best and potentially misleading at worst. We strongly encourage future investigations to incorporate multi-dimensional renal assessments to better inform clinical practice.
